# Survival outcomes of malignant peripheral nerve sheath tumors (MPNSTs) with and without neurofibromatosis type I (NF1): a meta-analysis

**DOI:** 10.1186/s12957-023-03296-z

**Published:** 2024-01-09

**Authors:** Zhixue Lim, Tian Yuan Gu, Bee Choo Tai, Mark Edward Puhaindran

**Affiliations:** 1 Department of Hand & Reconstructive Microsurgery, University Orthopaedic, Hand & Reconstructive Microsurgical Cluster, National University Health System, 1E Kent Ridge Road, NUHS Tower Block, Level 11, Singapore, 119228 Singapore; 2 Saw Swee Hock School of Public Health, National University of Singapore and National University Health System, 12 Science Drive, #10-01, Singapore, 117549 Singapore; 3 Yong Loo Lin School of Medicine, National University of Singapore, Singapore, Singapore

**Keywords:** Malignant peripheral nerve sheath tumors, Sarcoma, Neurofibromatosis, Survival, Outcomes, Mortality

## Abstract

**Introduction:**

Malignant peripheral nerve sheath tumors (MPNSTs) are malignancies that demonstrate nerve sheath differentiation in the peripheral nervous system. They can occur sporadically or be associated with neurofibromatosis type 1 (NF1), an autosomal dominant neurocutaneous disorder, with up to 13% of patients developing MPNSTs in their lifetimes. Previous studies have suggested conflicting findings regarding the prognosis of NF1 for patients with MPNSTs. The elucidation of NF1 as an independent prognostic factor on mortality has implications for clinical management. We aim to investigate the role of NF1 status as an independent prognostic factor of overall survival (OS) and disease-specific survival (DSS) in MPNSTs.

**Methods:**

An electronic literature search of PubMed and MEDLINE was performed on studies reporting OS or DSS outcomes of MPNSTs with and without NF1. A grey literature search by reviewing bibliographies of included studies and review articles was performed to find pertinent studies. Data was extracted and assessed in accordance with the PRISMA guidelines. A meta-analysis was performed to calculate hazard ratios (HRs) using a random-effects model. The primary and secondary outcomes were all-cause and disease-specific mortality, respectively, with NF1 as an independent prognostic factor of interest.

**Results:**

A total of 59 retrospective studies involving 3602 patients fulfilled the inclusion criteria for OS analysis, and 23 studies involving 704 MPNST patients were included to evaluate DSS outcomes. There was a significant increase in the hazard of all-cause mortality (HR 1.63, 95% CI 1.45 to 1.84) and disease-specific mortality (HR 1.52, 95% CI 1.24 to 1.88) among NF1 as compared to sporadic cases. Subgroup analyses and meta-regression showed that this result was consistent regardless of the quality of the study and year of publication.

**Conclusion:**

NF1 is associated with a substantially higher risk of all-cause and disease-specific mortality. This finding suggests that closer surveillance is required for NF1 patients at risk of developing MPNSTs.

**Supplementary Information:**

The online version contains supplementary material available at 10.1186/s12957-023-03296-z.

## Background

Malignant peripheral nerve sheath tumors (MPNSTs) are malignancies that show nerve sheath differentiation in the peripheral nervous system and have been previously described as neurogenic sarcoma, neurofibrosarcoma, malignant schwannoma, and malignant neurilemmoma [1]. They are rare with an incidence of 1.46 per million person-years in the general population [2]. In neurofibromatosis type 1 (NF1), an autosomal dominant neurocutaneous disorder, with an estimated birth incidence of 1 in 2500, up to 13% of patients develop MPNSTs in their lifetimes [3, 4]. These tumors tend to arise from pre-existing plexiform neurofibromas, which are uncommon in non-NF1 patients [1, 5].

It is unknown if sporadic MPNST (sMPNST) or NF1-related MPNST (nfMPNST) exhibit variable behavior, and there have been conflicting reports on factors predicting survival for patients with sMPNST and nfMPNST. The prognosis for MPNST is poor and has remained abysmal in the last few decades with 5-year survival rates ranging between 16 and 62% [6, 7]. Some studies have reported poorer survival rates in the NF1 group [4, 8–11], while others have suggested that there is little difference [12, 13]. Biologically, somatic changes in NF1, CDKN2A/B, and polycomb repressor complex 2 (PRC2) are identified in most MPNSTs regardless of NF1 status, and the set of genetic events leading to the development of NF1 associated and sporadic MPNSTs follow a similar pathogenic process [14, 15]. Therefore, the basis of NF1 as an independent (poor) prognostic factor in MPNST has been a constant source of contention. The clarification of NF1’s role in the prognosis of MPNST would enable clinicians to stratify the risk of the disease more effectively. This would have significant implications for staging, follow-up, and subsequent management. It is imperative, as MPNSTs are largely resistant to chemotherapy and radiotherapy, and a timely wide excision with clear margins remains the only possible curative option [16, 17]. In this meta-analysis, our aim is to investigate the impact of NF1 on the mortality of sMPNSTs compared to nfMPNSTs.

## Methods

### Literature search strategy

**Fig. 1:**
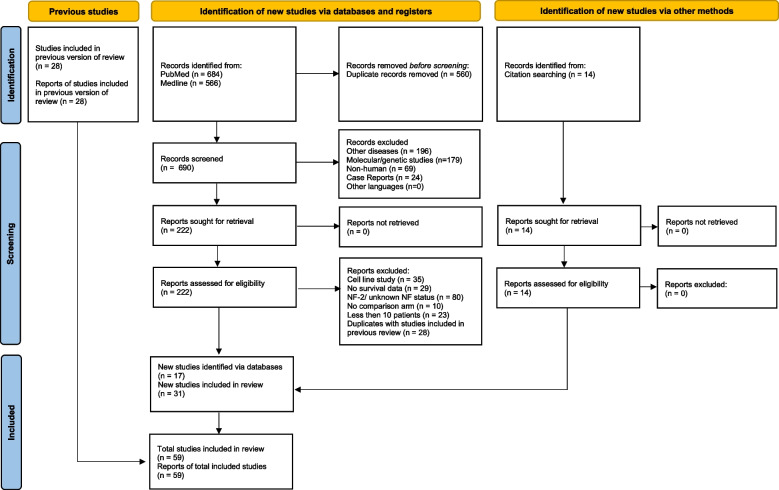
PRISMA flow chart

This meta-analysis was done in accordance with the Preferred Reporting Items for Systematic Reviews and Meta-Analyses (PRISMA) guidelines [18]. The review protocol was registered in the PROSPERO International Prospective Register of Systematic Reviews, registration number CRD42021275352, https://www.crd.york.ac.uk/prospero/display_record.php?ID=CRD42021275352. A search was conducted on PubMed and MEDLINE (via Ovid) up until September 30, 2021, with no language limitations. The search term employed was “peripheral nervous system neoplasms” [MeSH Terms] OR “nerve sheath neoplasms” [MeSH Terms] AND “malignant” AND “human” AND (prognosis OR mortality OR survival OR clinicopathologic). In addition to PubMed search results, we searched the grey literature by reviewing the bibliographies of the included studies as well as review articles. The reference lists of selected publications were reviewed to find pertinent studies, ensuring that the literature review was systematic and rigorous. The study protocol underwent revision due to the diverse formats of survival data presented in the studies. This required conducting different statistical analyses to extract as much survival data as possible from the existing literature. Figure 1 shows the flowchart of the literature review and study selection for this meta-analysis.

### Study selection

Studies reporting overall survival (OS) or disease-specific survival (DSS) outcomes of malignant peripheral nerve sheath tumors with and without NF1 in humans were included. The inclusion criteria were (i) clear diagnosis of NF1 status, (ii) two-arm study involving sMPNSTs and nfMPNSTs, (iii) more than ten patients in each study, (iv) sufficient statistical information to allow quantification of effect based on hazard ratio (HR), and its 95% confidence interval (CI). We set a minimum of ten patients per study as a cutoff to balance the prospect of achieving a reliable survival estimate for hazard ratio analyses and reduce the risk of excluding too many studies given that most study samples are small. Review articles, case reports, animal studies, cell line studies, molecular/genetic/cellular markers studies, and duplicated studies were excluded. As in the previous literature [19], six articles [14, 20–24] were combined according to the same first author to form three distinct studies in our meta-analysis, after verifying that there was no duplication. For three further studies, [9, 25, 26] only the latest published result was included since the prior two studies were incorporated in the last publication.

### Data extraction

Data extracted included the name of the first author, publication year, country, study size per arm and overall, age, gender, anatomical location, and median duration of follow-up. For papers that did not report the mean age and its variance, the mean and SD were calculated using the methods described by Hozo et al. [27]. The assessment of the quality of studies was evaluated using the Newcastle–Ottawa Scale (NOS) by two different reviewers (ZL, MB), and disagreements were resolved by consensus or appeal to a third author (MP) [28]. The total quality score ranged from 0 to 9, contributed by three components including selection of the exposed and unexposed cohort, comparability of the groups, and outcome assessment. Studies that scored 7–9, 4–6, and 3 or fewer points were classified as having a low, moderate, or high risk of bias, respectively.

### Statistical analysis

Publication bias was evaluated by visual examination of funnel plot of the standard error of log(HR) against log(HR), Egger’s asymmetry test, and the trim-and-fill method [29].

For studies which provided individual patient data (IPD), the HR and SE were estimated via the Cox proportional hazard model. For studies in which the number of events, log-rank test *p*-value, randomized number in each group, and confidence interval of HR were provided, the HR and SD were calculated according to the methodology described by Parmar et al., using the formula ${\text{exp}}[\left({{\text{O}}}_{{\text{ri}}}-{E}_{ri}\right)/{V}_{ri}]$ [30]. For studies which reported the 3-year or 5-year survival rate digitally or graphically, the pooled HR was estimated using the formulae of Tierney et al., assuming constant censoring within each interval [31]. If the median survival time for each arm was available only, the HR was estimated based on the ratio of median survival time [32]. For studies which provided the Kaplan–Meier (KM) curves only, the individual patient data were reconstructed from the KM curves using the methodology described by Guyot et al., and the HR was estimated via the Cox proportional hazard model [33]. When zero event was recorded in either arm, a continuity correction factor of 0.5 was applied to the number of events and non-events in each arm [34].

The DerSimonian-Laird random-effects model was implemented to derive the pooled estimate of HR and its corresponding 95% confidence interval [35]. This model assumed that the individual studies were drawn at random from a larger population, with each having its own underlying effect size. The interstudy heterogeneity is represented by *I* [2, 36]. We have further performed a sensitivity analysis excluding studies with confidence intervals (CI) larger than 100 to avoid overestimating the pooled estimate.

Further, meta-regression analyses were conducted based on publication year, NOS scores, and prespecified anatomical location (extremity versus non-extremity) to fully utilize the information by considering the entire range of the data, rather than dichotomizing them as binary variables based on pre-specified cutoffs. Besides, subgroup analysis was performed based on pre-specified anatomical location. As many studies have provided information for both extremity and non-extremity locations, the robust variance estimate was utilized to account for the non-independent effect sizes in the meta-regression based on anatomical location.

All statistical analyses were conducted based on a two-sided test assuming a 5% significance level, using STATA version 17.

## Results

### Characteristics of studies

These 59 studies selected were all retrospective studies published from 1963 to 2020. More than half were published after 2000 (61%), while 21 (36%) were published after 2010 (Table 1). Most studies (75%) were conducted in the USA or Europe, while studies from Asia were published mostly after 2010. The size of the study varied from 11 to 294 with 16 studies (27%) of size less than 20. The median follow-up duration ranged from 5 to 180 months. The mean age of patients in these studies ranged from 9.5 to 59 years. All studies reviewed were in the English language.

**Table 1:** Characteristics of studies reporting survival data for nfMPNSTs and sMPNSTs

First author	Published year	Country	Total no. of patients (analyzed)	No. of patients in NF1 arm/sporadic arm	Events	No. of gender female/male	Mean age in years (SD)	Median follow-up in months (range)
Angelov	1998	Toronto, Canada	18	7/11	7	10/8	45.9 (21.4)	67 (9–81)
Ariel	1993	New York, USA	74	30/44	48			
Arpornchayanon	1984	Tokyo, Japan	16	2/14	5	8/8	40.94 (9.3)	(7–144)
Bergamaschi	2017	Italy	73	35/38	60	36/37	≤ 10 years: 27 (37%) > 10 years: 46 (63%)	180 (42–430)
Bojsen-Moller	1984	Denmark	29	8/21	20	13/16	40.34 (19.43)	60 (24–240)
Brekke	2009–2010	Norway, Sweden, Netherlands	82	37/45	57	38/44	40.6 (20.7)	42 (1–369)
Carli	2005	Italy, Germany	167	29/138	89	84/83	10.5 (5.83)	87.6 (28.5–346.8)
Casanova	1999	Italy	24	7/17	16	11/13	10.06 (4.99)	36 (6–250)
Cashen	2004	USA	80	18/62	15	41/39	36 (17)	19.7 (8–276)
D’Agostino	1963	USA	41	19/22	30	21/20	37.8 (14.9)	21 (3–192)
Daimaru	1985	Japan	28	11/17	18	19/9	40.25 (18.59)	36 (3––204)
De Cou	1995	USA	27	11/16	15	14/13	13.89 (4.57)	25 (3–180)
De Vasconcelos	2017	Brazil	92	41/51	53	51/41	43.5 (23.77)	24.8 (2–252) for NF1, 46.5 (3–208) for sporadic^a^
Demir	2012	Turkey	13	4/9	8	6/7	10.2 (4.79)	8.5 (1–130)
Doorn	1995	Netherlands	22	11/11	16	8/14	39.5(22.99)	24 (1–153)
Ducatman	1986	USA	120	62/58	88	68/52	34 (20.29)	25.8 (0.11–120)
Evans	2002	UK	61	24/37	51		49.44 (20.65)	34.6 (11.1–204)
Fan	2013	China	146	17/129	53	67/79	< 40 years: 71 (49%) ≥ 40 years: 75 (51%)	
Ganju	2001	USA	12	5/7	7	3/9	40.9(19.6)	54 (6–119)
Guellec	2016	France	106	68/38	49	41/65	45.25 (24.2)	66 (39–90)
Halling	1996	USA	28	14/14	22		39.2 (17.4)	26 (1.5–251)
Hagel	2007	Germany	52	38/14	34	24/28	36.3 (17.1)	
Holtkamp	2007	Germany	35	22/13	20	17/18	39.4 (19.6)	29 (3–200)
Hong	2017	Korea	11	8/3	2	4/7	9.5 (6.04)	136.6 (2.3–167.1)
Hruban	1990	USA	43	23/20	30	22/21	40 (16.7)	31 (2–324)
Hwang	2017	Korea	95	33/62	57	45/50	42.38 (21.18)	166.76 (5.94–329.53)
Kamran	2012	USA	84	24/60	39	37/47	43.2 (22.7)	19 (0–329)
Kar	2006	India	24	5/19	10	5/19	44.3 (18.8)	38^a^
Kourea	1998	USA	25	15/10	20	12/13	31.6 (12.2)	11 (2–221)
Kunisada	1997	Japan	11	5/6	9	4/7	44.5 (17.57)	15 (2–52)
Lamm	2013	Austria	14	6/8	10	7/7	50.25 (18.72)	41 (1–108)
Lodding	1986	Sweden	14	1/13	10	9/5	42.14 (17.6)	24 (2–216)
Longhi	2010	Italy	62	22/40	40	23/39	44 (18.5)	54 (12–194)
Loree	2000	USA	17	7/10	10	8/9	41.25 (22.4)	139.2 (40.8–432)
Martin	2020	USA	70	26/44	25	32/38	All ≤ 18 years	(1.4–150)
McCarron	1998	USA	15	12/3	7	7/8	38(21.03)	16 (3–120)
McCaughan	2007	Scotland	25	12/13	17		59 (21.2)	31.12 (0.9–139.55)
Meis	1992	USA	57	13/44	28	26/31	< 7 years: 23 (40%) ≥ 7 yeas: 34 (60%)	22 (2–230)
Mertens	2000	Sweden	19	7/12	12	9/10	34.53 (16)	36 (7–84)
Miao	2019	USA	259	77/182	223	132/127	40.5 (27.5)	75.87 (0.28–378.22)
Okada	2007	Japan	56	25/31	32	34/22	45 (20.57)	44.6 (2–156)
Owosho	2018	USA	13	9/4	8	6/7	30.8 (16)	24 (13–141)
Porter	2009	UK	123	33/90	56		Median: NF1 26, sporadic 53	(6–252)
Raney	1987	USA	24	16/8	14	14/10	10.56 (5.53)	17 (4–291.6)
Scheithauer	2009	USA	12	4/8	11	2/10	41.5 (15.2)	5 (1.5–180)
Schmidt	1999–2000	Germany	26	6/20	13	14/12	48.27 (16.8)	27 (2–168)
Sordillo	1981	USA	165	65/100	104	89/76	41.4 (26.3)	
Tabone-Eglinger	2008	France	52	26/26	23	22/30	23 (15)	
Trojanowski	1980	USA	18	2/16	8	13/5	48.3 (19)	54 (1–90)
Valentin	2016	France	294	106/188	182			87.6 (2.08–279.1)
Van Noesel	2019	Netherlands	51	25/26	20	26/25	12.2 (6.1)	64.6 (1.3–147.7)
Wang	2013	China	13	3/10	2	5/8	57.2 (16.8)	(4–132)
Wang	2015	China	43	6/37	22	18/25	< 50 years: 23 (53%) ≥ 50 years: 20 (47%)	24 (2–115)
Watson	2004	USA	40	24/16	26	23/17	38.48 (15.17)	18 (2–126)
White	1971	USA	15	10/5	9	7/8	36.87 (11.59)	16 (2–156)
Wong	1998	USA	134	32/102	69	52/82	41.75 (22.03)	53 (7–280)
Yu	2011	USA	122	47/75	88	67/55	38.25 (18.98)	18 (1–180)
Yuan	2017	China	159	70/89	101	78/81	40.25 (20.69)	31 (2–199)
Zhou	2016	USA/China	51	35/16	23	22/29	≥ 40 years: 22 (43%) < 40 years: 29 (57%)	

^a^Mean is presented in this study

### Study quality

The median NOS score was 7 (range 4 to 8), with the majority of the studies having a score of at least 7 (72%) suggesting a low risk of bias. For 16 studies with moderate risk of bias, they were largely due to ambiguity in follow-up duration or not accounting for participants who were lost to follow-up.

### Assessment of publication bias

The funnel plot (Additional file 1: Fig. S1) tended to be symmetrical about the pooled log(HR) of 0.48. In addition, Egger’s test revealed no evidence of small-study effects (*p* = 0.799). However, there was some evidence of heterogeneity between studies ${(I}^{2}=29\%;p=0.02).$ The funnel plot after applying the trim-and-fill method (Additional file 2: Fig. S2) is consistent with the previous observation, and it tended to be symmetrical about the log(HR). The mean effect size based on 59 studies is 0.49 (95% CI 0.37 to 0.61), and the updated estimate based on 62 studies is 0.48 (95% CI 0.36 to 0.60).

### Meta-analysis of 59 articles (OS) and 23 articles (DSS)

**Fig. 2:**
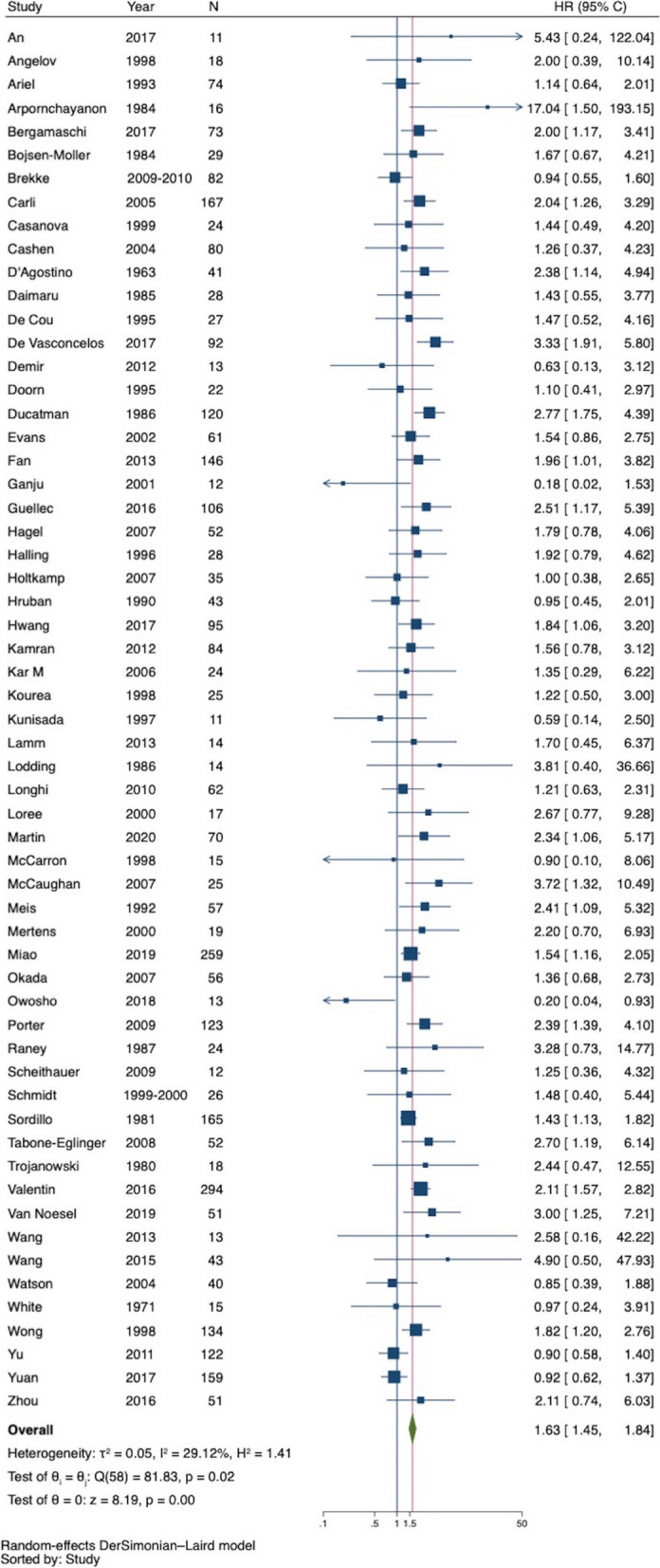
Forest plot of all-cause mortality of NF1 versus non-NF1 patients

The 59 studies included in the meta-analysis for OS involved a total of 3602 patients and 2141 events [4, 8–14, 20, 21, 23, 24, 37–85]. There were 1361 patients in the nfMPNST arm and 2241 patients in the sMPNST arm. The HR of individual studies ranged from 0.18 to 17.04, and the pooled random effect estimate was 1.63 (95% CI 1.45 to 1.84) (Fig. 2). The exclusion of 2 studies with CI > 100, (Arponchayon et al. 1984 and An et al. 2017) did not affect the effect estimate, HR = 1.62 (92% CI 1.44–1.82).

**Fig. 3:**
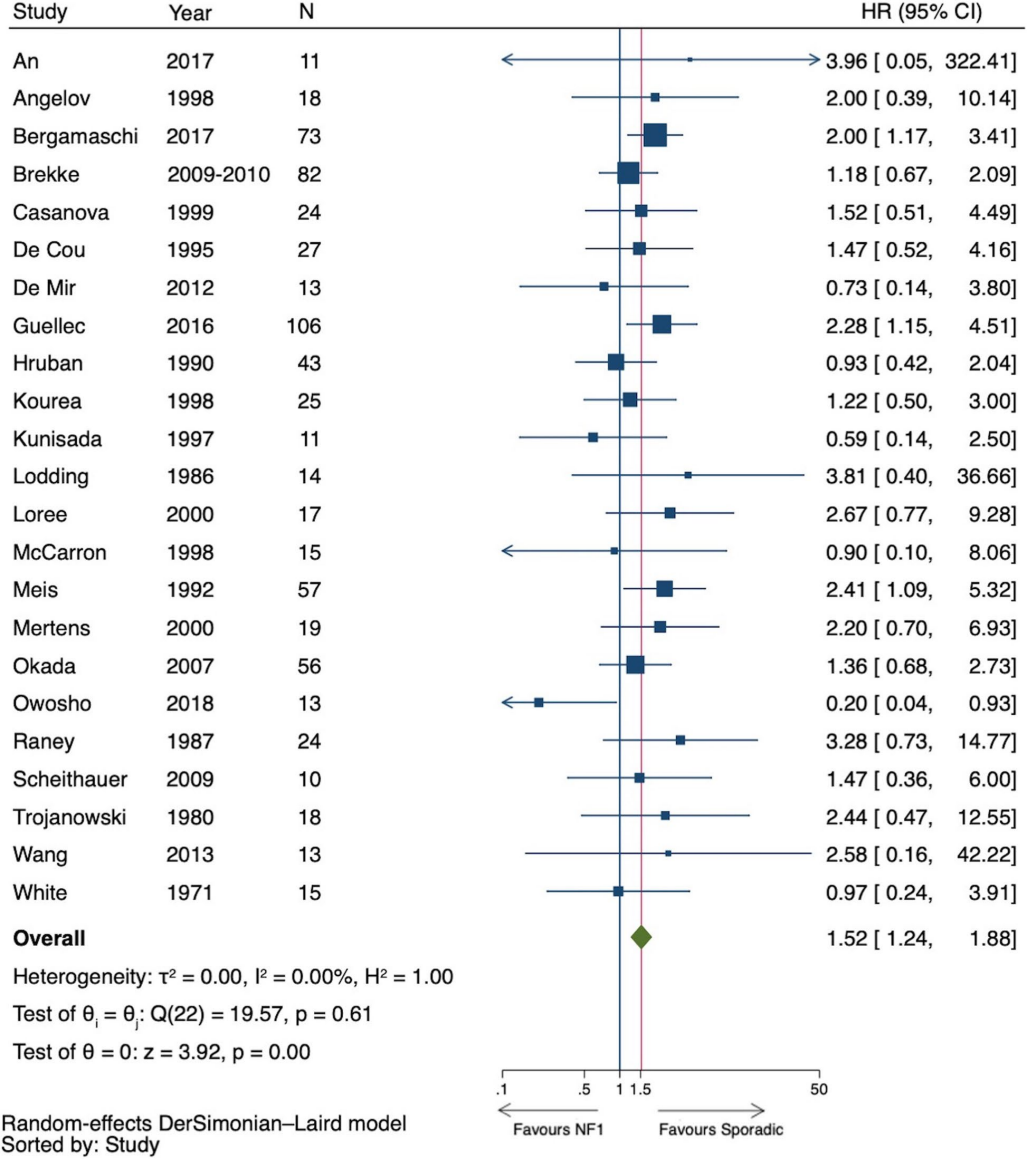
Forest plot of disease-specific mortality of NF1 versus non-NF1 patients

The 23 studies included in the meta-analysis for DSS involved a total of 704 patients and 393 events [12, 20, 37, 40, 43, 45, 47, 51, 55, 59, 60, 62, 64, 66, 68, 69, 71–74, 76, 79, 82]. There were 330 patients in the nfMPNST arm and 374 patients in the sMPNST arm. The HR of individual studies ranged from 0.20 to 3.96, and the pooled random effect estimate was 1.52 (95% CI 1.24 to 1.88) (Fig. 3). The exclusion of a study with CI > 100 (An et al. 2017) did not affect the effect estimate, HR = 1.52 (95% CI 1.23– 1.88).

#### Meta-regression by publication year

Twenty-three (39%) studies involving a total of 974 patients were published before 2000, with sample sizes ranging from 11 [60] to 165 [75]. Thirty-six studies including a total of 2628 patients were published post-2000, with study size ranging from 11 [55] to 294 [77]. The meta-regression suggested that the effect of NF1 on OS did not change according to the year of publication (*β* =  − 0.001, 95% CI − 0.010 to 0.008, *p* = 0.881) (Additional file 3: Fig. S3). Similarly, the meta-regression suggested that the effect of NF1 on DSS did not change according to the year of publication (*β* = 0.001, 95% CI − 0.017 to 0.020, *p* = 0.895) (Additional file 4: Fig. S4).

#### Meta-regression by NOS score

There were 43 studies with scores ≥ 7 involving 2823 patients and 16 studies involving 779 patients with scores less than 7. The regression coefficient for the NOS score was 0.02 (95% CI − 0.09 to 0.13, *p* = 0.705), suggesting that the effect of NF1 on OS was relatively unaffected by the NOS score (Additional file 5: Fig. S5). The regression coefficient for the NOS score on DSS was − 0.09 (95% CI − 0.29 to 0.11, *p* = 0.386), suggesting that the effect of NF1 on DSS was also relatively unaffected by the NOS score (Additional file 6: Fig. S6).

#### Subgroup analysis and meta-regression by anatomical location

**Fig. 4:**
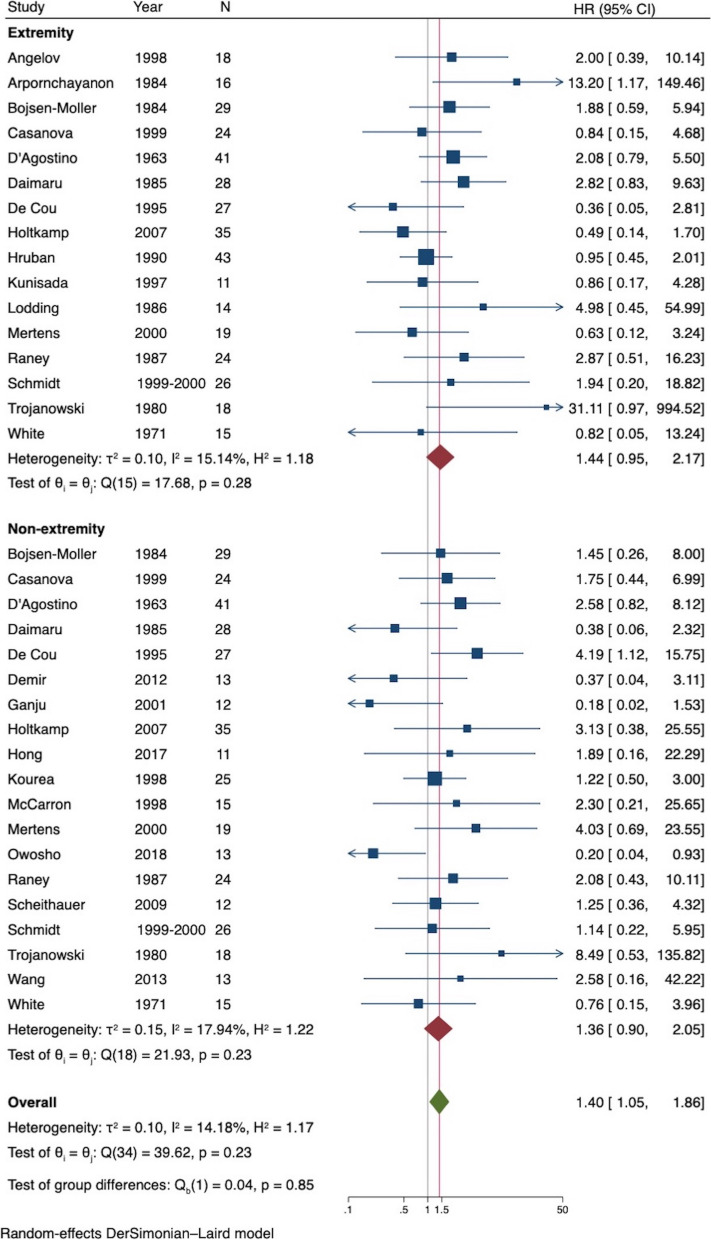
Forest plot of all-cause mortality of NF1 versus non-NF1 patients by anatomical location

**Fig. 5:**
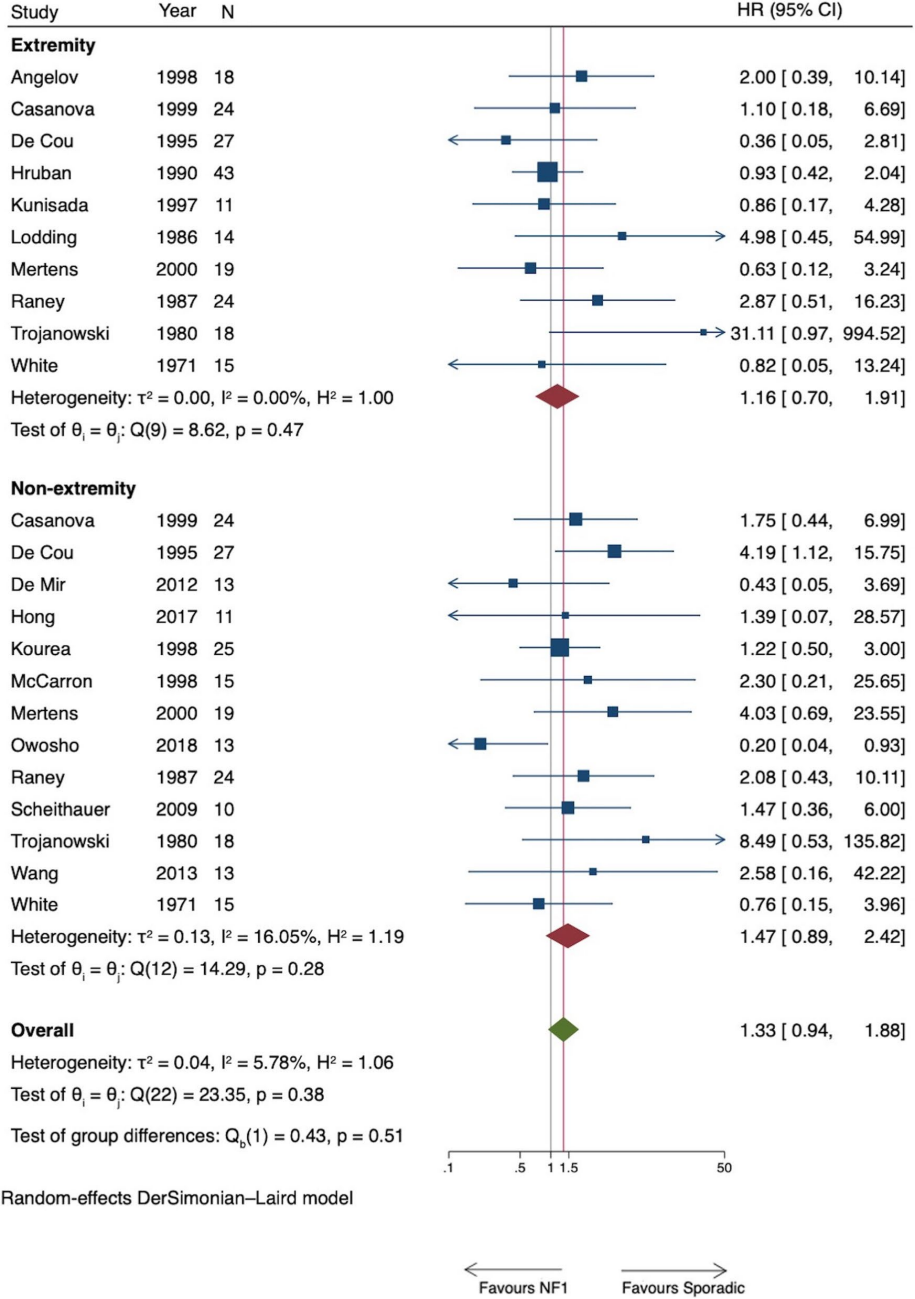
Forest plot of disease-specific mortality of NF1 versus non-NF1 patients by anatomical location

A total of 24 studies provided information for subgroup analysis based on anatomical location. Of these, 11 studies included both extremity and non-extremity locations, 5 studies had information for only extremity location, and 8 studies provided information for only non-extremity locations. A total of 388 patients with extremity and 400 patients with non-extremity location were thus included. The HR for the analysis of OS ranged from 0.36 to 31.11 for the 16 studies that reported extremity location and 0.18 to 8.49 for the 19 studies that reported non-extremity locations. There was no difference in HR of all-cause mortality between studies that reported extremity (HR = 1.44, 95% CI 0.95 to 2.17) and those that reported non-extremity (HR = 1.36, 95% CI 0.90 to 2.05) (*p* = *0.847*) (Fig. 4). The effect estimate of location based on meta-regression was 1.04 (95% CI 0.53 to 2.03, *p* = 0.906). The HR for the analysis of DSS ranged from 0.63 to 31.11 for the 10 studies that reported extremity location and 0.20 to 8.49 for the 13 studies that reported non-extremity locations. There was also no difference in HR of disease-specific mortality between studies that reported extremity (HR = 1.16, 95% CI 0.70 to 1.91) and those that reported non-extremity (HR = 1.47, 95% CI 0.89 to 2.42) (*p* = 0.51) (Fig. 5). The effect estimate of location based on meta-regression was 0.80 (95% CI 0.36 to 1.82, *p* = 0.568).

## Discussion

MPNSTs are associated with the highest risk of mortality among soft tissue sarcomas [86]. Due to its rarity, there are few large studies on factors predicting survival. Among these factors, there is a lack of consensus on whether NF1 is an independent indicator of poor prognosis. In this present meta-analysis of 3602 individuals with MPNSTs, we found that NF1 was associated with a 63% increase in the hazard of all-cause mortality and a 52% increase in the hazard of disease-specific mortality compared to sporadic cases. This is similar to the findings by other authors which have reported poorer prognoses for NF1 patients [9, 87], with some demonstrating worse survival outcomes in the NF1 group despite comparable histological grades and tumor sizes [8, 16, 75].

However, there had been previous studies that reported no difference in survival outcomes of MPNST between NF1 and non-NF1 patients [12, 13, 88]. In a large MPNST meta-analysis, Kolberg et al. proposed the notion that the survival difference between NF1 and sporadic MPNSTs was diminishing with a trend towards improved survival in NF1 patients with additional data from more recent publications [19]. To better evaluate the association of survival data with the period of publication, we conducted a meta-regression by year of publication. Our results did not support this notion as we did not detect any difference in the effect of NF1 on all-cause mortality across the years of publication. This can be explained by the lack of novel therapeutic developments for MPNSTs in the past several decades as they remain largely resistant to chemotherapy and radiotherapy [86]. To date, no molecular targeted therapy has demonstrated efficacy in the reduction of tumor size or improvement in survival outcomes [89]. Complete surgical extirpation with clear margins remains the only potential curative therapy for MPNST [17]. The quantification of effect estimates of our study based on HR compared to odds ratio (OR) in Kolberg’s study could also contribute to our varied conclusions. We opted to use HR analysis as this fully utilizes the available information on when a MPNST mortality occurs, instead of simply considering whether an event occurs in an OR analysis.

Given that NF1 has been established as a significant predictor of mortality, we propose considering NF1 status when staging MPNSTs and advocate closer monitoring of these patients. It is imperative that health systems take extra measures to ensure that NF1 patients have prompt and convenient access to specialized care, particularly if they have suspicions of malignancies. Furthermore, we recommend that clinicians lower their threshold in obtaining advanced imaging to facilitate early detection of tumors in this (NF1) population.

While we conclusively show that NF1 is a significant poor prognostic factor in MPNST, the reason for this remains unanswered. From a biological standpoint, suggestions that NF1 tumors are inherently more aggressive have been disputed as the genomic aberrations in NF1, CDKN2A, Polycomb repressive complex 2 (PRC2), PDGFRA, EGFR, MET, and TP53 found in both nfMPNST and sMPNST are largely similar [90] (p2), [91]. On the other hand, there is data to suggest that tumorigenesis begins earlier for NF1 patients, and these tumors are more resistant to treatment, leading to poorer outcomes [42]. We postulate that while the genomic aberrations are similar even for MPNSTs of divergent differentiation, the precise sequence of pathogenic events may be crucial in our understanding of the disease [91, 92]. The findings of our study suggest that NF1 patients require closer clinical follow-up to diagnose and treat these aggressive tumors early.

In terms of anatomical sites of presentation, they are largely similar for both sporadic and NF1 patients [9]. Some studies have reported better survival outcomes for MPNSTs in distal extremities [75, 88], as well as lower rates of recurrence [88]. As part of our study, we did a subgroup analysis to distill the effect of anatomical location on clinical outcomes. We found no significant difference in OS and DSS for extremity and non-extremity MPNSTs. We postulate that the prognostic value of the anatomical site of the disease may not be as important as attaining a negative margin, which has been proven to be an important factor in predicting local recurrence and survival among patients with soft tissue sarcomas [93–97]. Another plausible explanation is the relative ease of achieving a negative resection margin in the extremity and the additional benefit of clinical surveillance for tumors of the extremities, where local recurrences may be more apparent without the need for image-guided surveillance.

Our study has several limitations and caveats. First, it is important to recognize limitations with MPNST being a rare condition, and there is a lack of standardization in terms of follow-up and data collection for the studies. The rarity of MPNSTs explains the heterogeneity seen in our forest plots, like in previous analyses. Second, we acknowledged that NF1 patients are vulnerable to other causes of mortality apart from MPNSTs and hence included DSS analysis to reduce potential biases. A previous large meta-analysis of > 1800 patients has found no discernible difference between overall survival and disease-specific survival when comparing NF1 and sporadic cases of MPNST [19]. This is in contrast to our findings where NF1 was associated with an increased hazard of both all-cause and disease-specific mortality. Third, it is crucial to recognize that the treatment regime for each study was different with some offering adjuvant chemoradiotherapy as an option. However, this information was often not provided in these studies to allow for adjustment. Fourth, all our studies are retrospective and observational in nature. The intrinsic biases of observational studies such as loss to follow-up and confounders hence cannot be eliminated. Subgroup analyses that we have performed may also have limited power to detect the differences due to small numbers. However, it is unlikely that a randomized controlled trial with sufficient power can be established given the low incidence of MPNSTs.

## Conclusion

The results of this study show that NF1 is associated with a substantially higher risk of all-cause and disease-specific mortality for MPNSTs. We found no significant change in the association of NF1 as a poor prognostic indicator of mortality with time of publication or with the anatomical location of the tumor. As such, clinicians should consider NF1 status in staging the disease and closer monitoring of NF1 patients at risk of developing MPNSTs to enhance their survival rates through timely intervention.

### Supplementary Information

**Additional file 1: Fig. S1.** Funnel plot for publication bias.

**Additional file 2: Fig. S2.** Funnel plot for publication bias after trim-and-fill method.

**Additional file 3: Fig. S3.** Meta-regression: logHR of OS on year of publication.

**Additional file 4: Fig. S4.** Meta-regression: logHR of DSS on year of publication.

**Additional file 5: Fig. S5.** Meta-regression: logHR of OS on NOS scores.

**Additional file 6: Fig. S6.** Meta-regression: logHR of DSS on NOS scores.

## Data Availability

The datasets used and/or analyzed during the current study are available from the corresponding author upon reasonable request.

## Abbreviations

MPNST: Malignant peripheral nerve sheath tumors
NF1: Neurofibromatosis type I
sMPNST: Sporadic MPNST
nfMPNST: NF1-related MPNST
KM: Kaplan-Meier
OS: Overall survival
DSS: Disease-specific survival
NOS: Newcastle-Ottawa Score
HR: Hazard ratio
OR: Odds ratio
PRISMA: Preferred Reporting Items for Systematic Reviews and Meta-Analyses
IPD: Individual patient data
