# Supplementary figures and images for: Survival outcomes of malignant peripheral nerve sheath tumors (MPNSTs) with and without neurofibromatosis type I (NF1): a meta-analysis
Source: World J Surg Oncol. 2024 Jan 9;22:14. doi: 10.1186/s12957-023-03296-z (PMC10775467; doi:10.1186/s12957-023-03296-z)

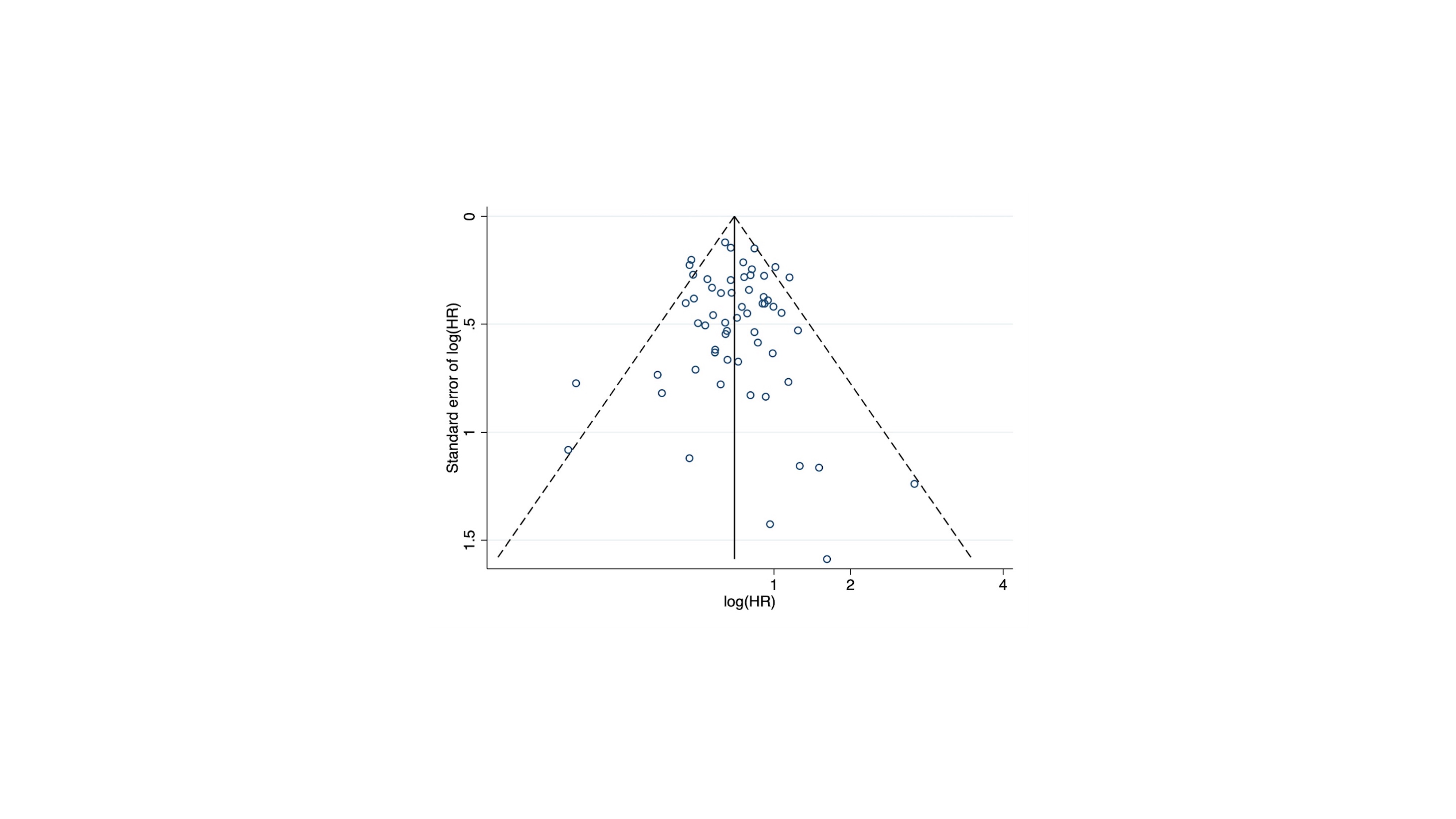

Supplement: Supplementary file 1 — Additional file 1: Fig. S1. Funnel plot for publication bias. [file 12957_2023_3296_MOESM1_ESM.jpg]

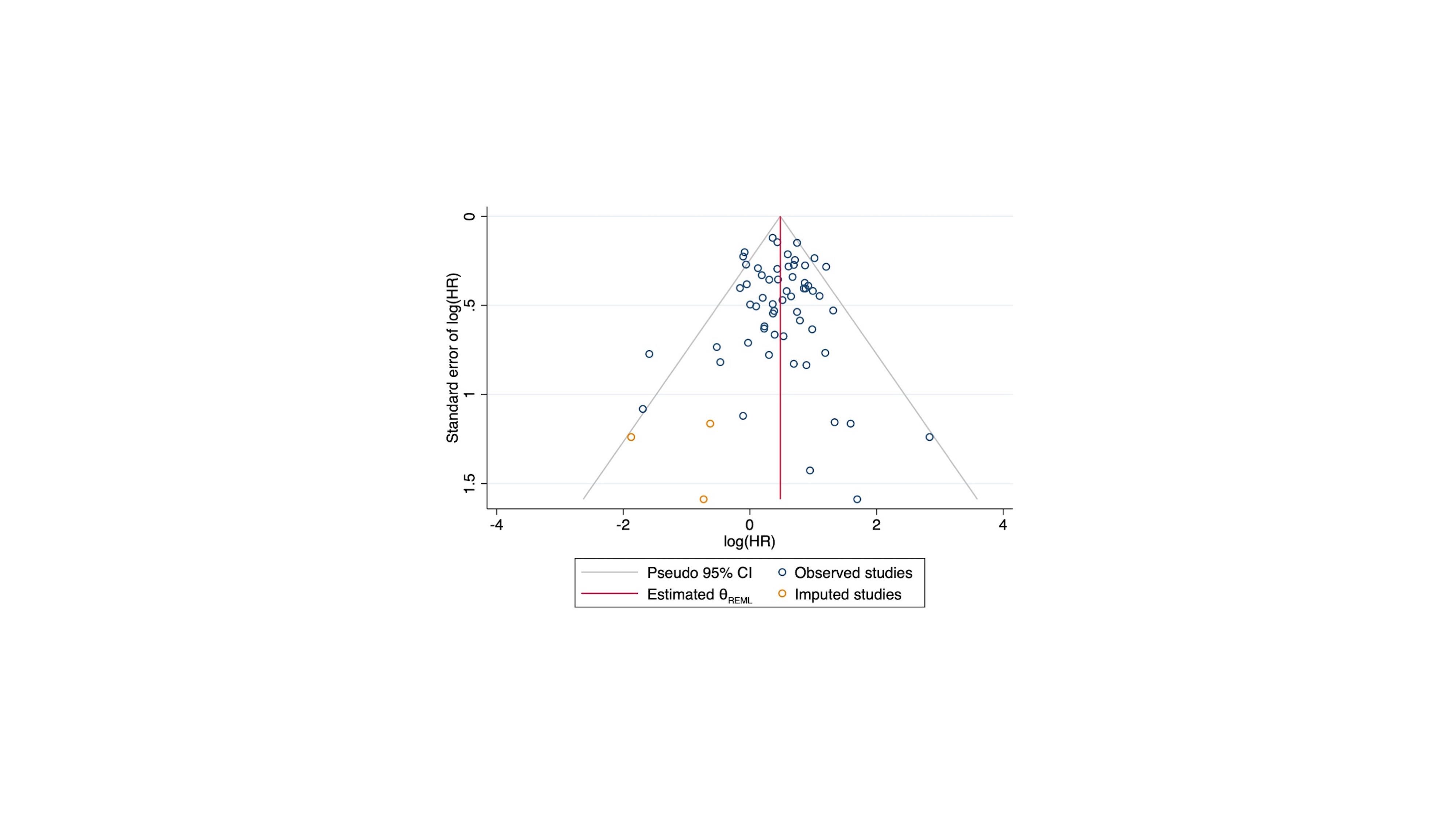

Supplement: Supplementary file 2 — Additional file 2: Fig. S2. Funnel plot for publication bias after trim-and-fill method. [file 12957_2023_3296_MOESM2_ESM.jpg]

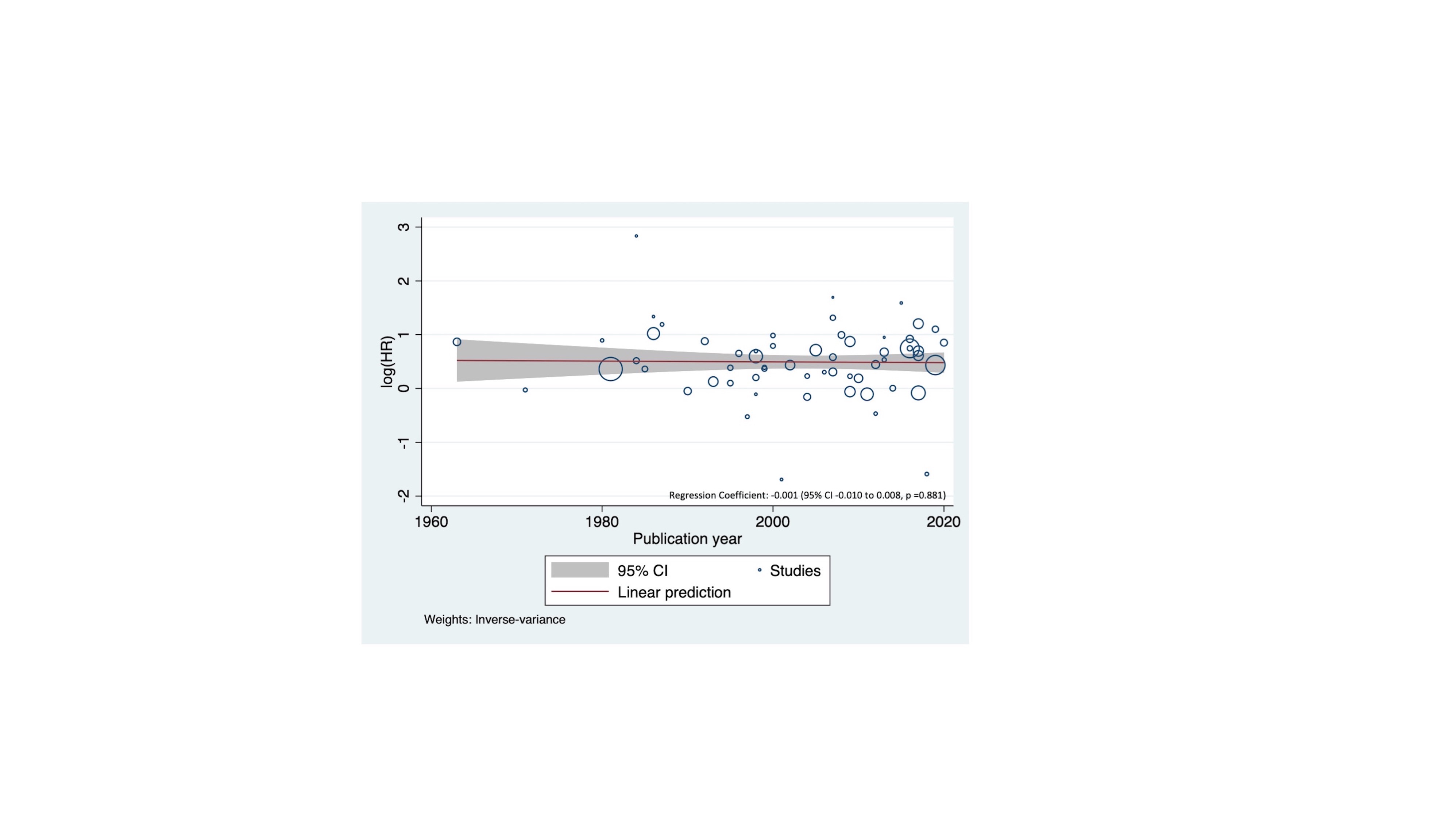

Supplement: Supplementary file 3 — Additional file 3: Fig. S3. Meta-regression: logHR of OS on year of publication. [file 12957_2023_3296_MOESM3_ESM.jpg]

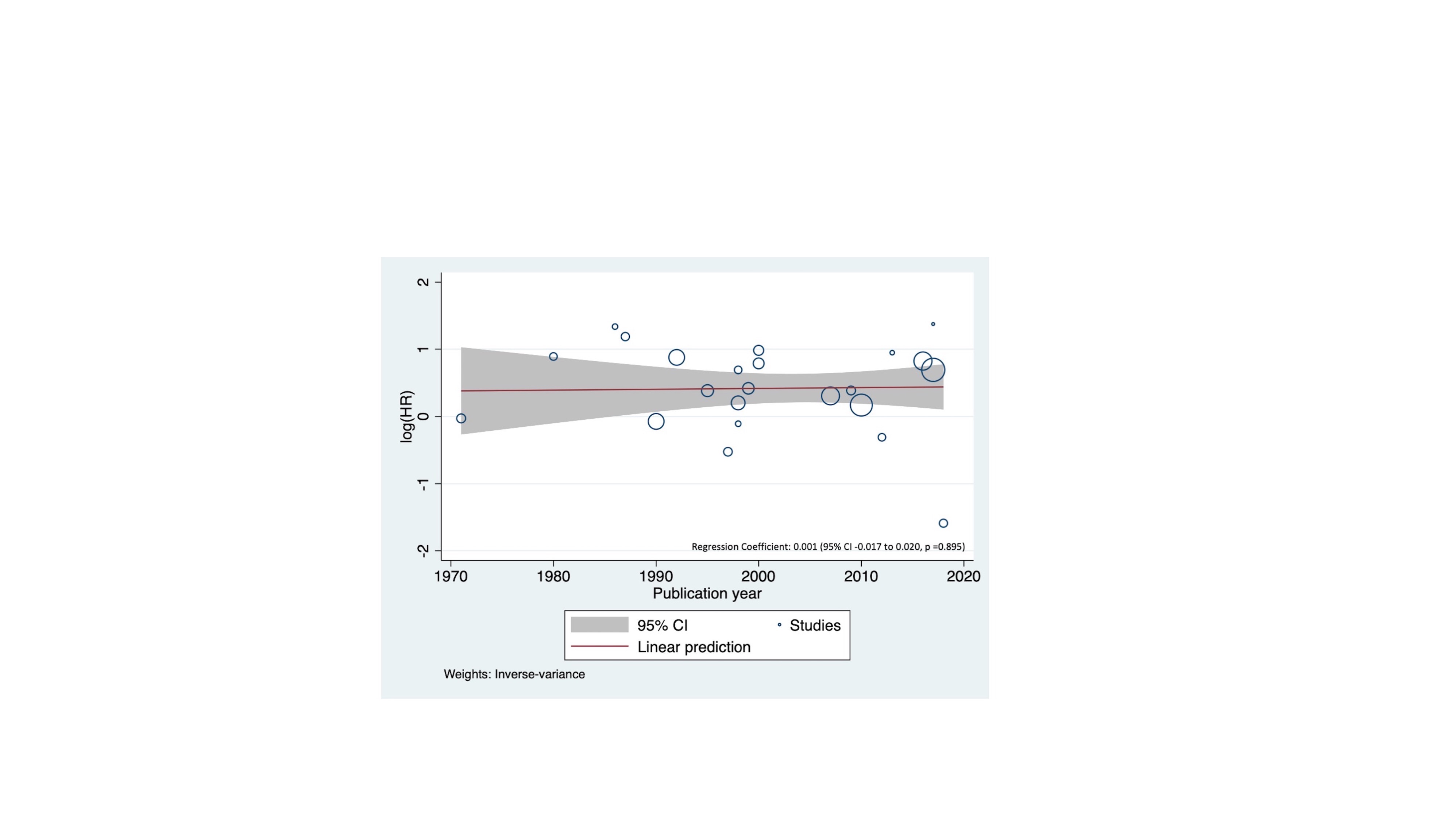

Supplement: Supplementary file 4 — Additional file 4: Fig. S4. Meta-regression: logHR of DSS on year of publication. [file 12957_2023_3296_MOESM4_ESM.jpg]

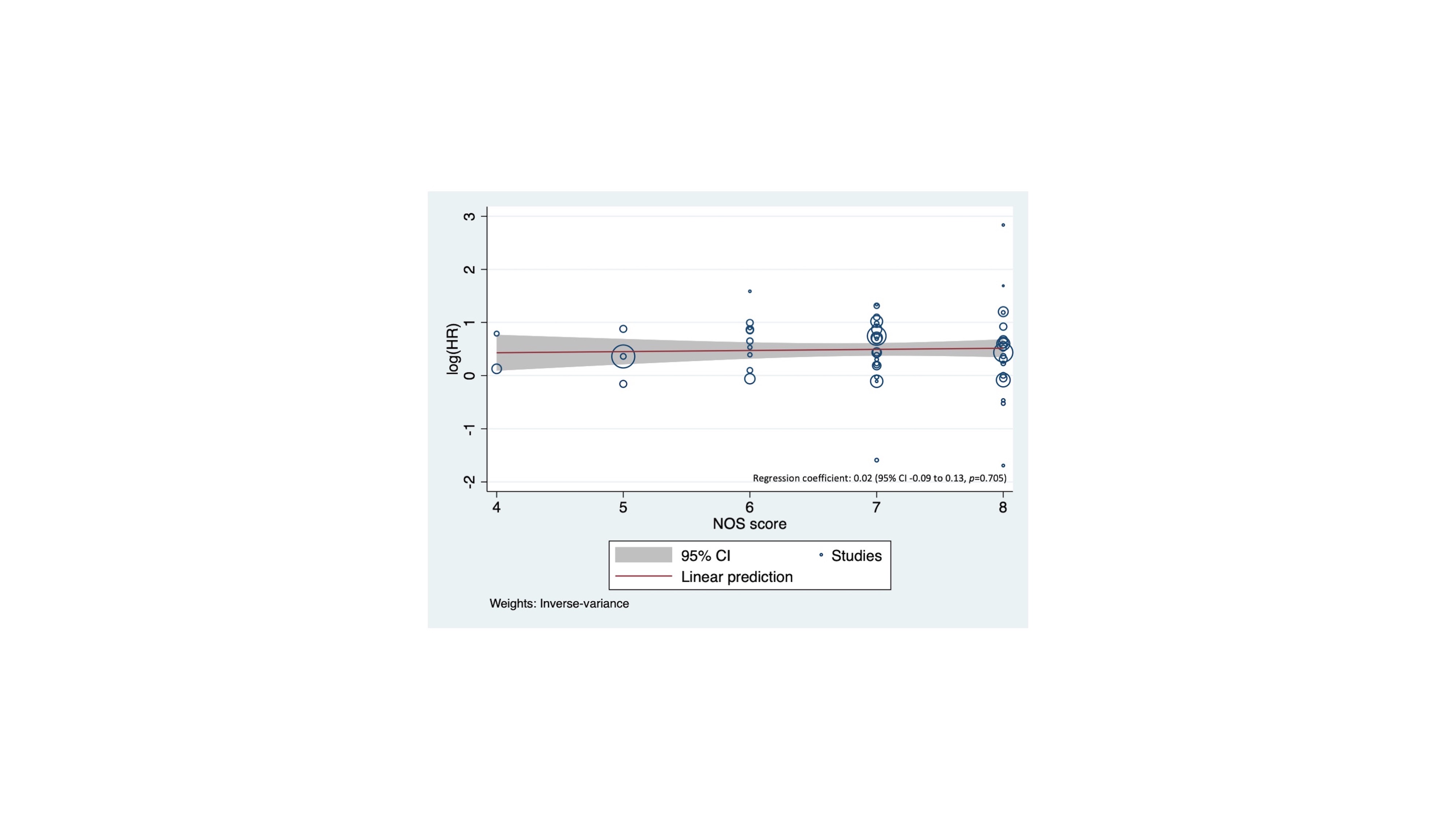

Supplement: Supplementary file 5 — Additional file 5: Fig. S5. Meta-regression: logHR of OS on NOS scores. [file 12957_2023_3296_MOESM5_ESM.jpg]

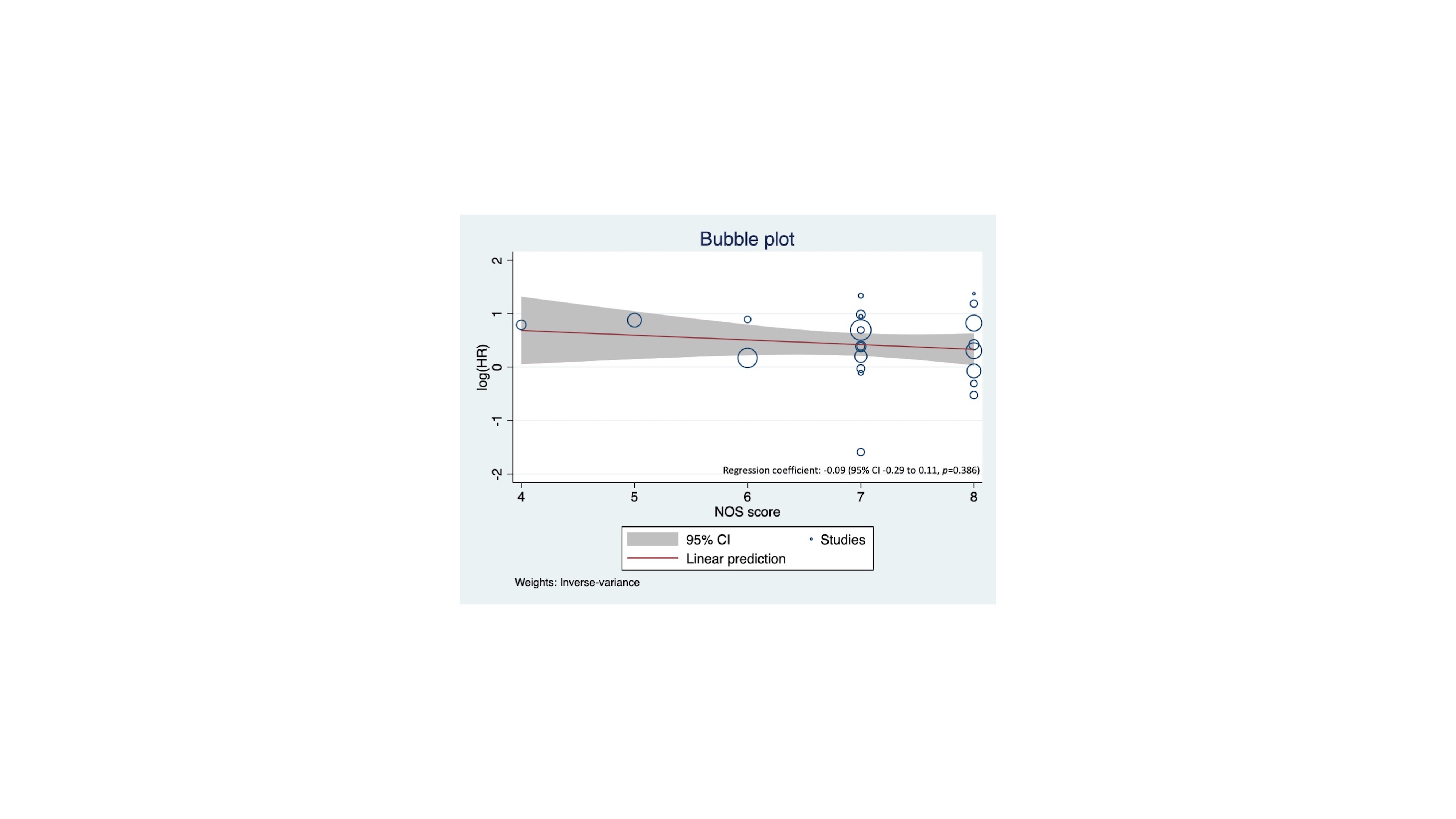

Supplement: Supplementary file 6 — Additional file 6: Fig. S6. Meta-regression: logHR of DSS on NOS scores. [file 12957_2023_3296_MOESM6_ESM.jpg]
